# Bounded personality diversity boosts collective intelligence: a swarm optimization approach

**DOI:** 10.3389/fpsyg.2026.1774466

**Published:** 2026-07-20

**Authors:** Todd A. Pringle, Lyle Stramer, Michael D. Robinson

**Affiliations:** Department of Psychology, North Dakota State University, Fargo, ND, United States

**Keywords:** collective cognition, diversity, evolution, particle swarm optimization, personality, swarm intelligence

## Abstract

**Introduction:**

This study presents an initial computational test of bounded diversity advantage theory (BDAT), which posits that bounded personality diversity enhances emergent collective problem-solving, using a Particle Swarm Optimization (PSO) testbed.

**Methods:**

The current PSO instantiation includes a novel monitoring approach to quantify group agreement dynamics. Focusing on conscientiousness-like behavior, operationalized as industriousness and persistence in swarm agents through adaptive inertia, six diverse and non-diverse swarm compositions were compared across 11 two-dimensional mathematical search functions. Swarm outcomes were evaluated using a Performance Efficiency Score (PES), which balances error (fitness) and iterations (time).

**Results:**

Diverse compositions outperformed non-diverse compositions, consistent with the present operationalization of BDAT-derived predictions concerning bounded diversity and premature closure. Monitoring asymmetry in polarized swarms at the first convergence threshold (50%) further indicated that highly industrious agents disproportionately drove early convergence while low-industriousness agents remained more exploratory, a process pattern also consistent BDAT predictions.

**Discussion:**

These findings suggest that PSO can serve as a useful computational testbed for examining some BDAT mechanisms, while broader validation of BDAT will require additional research. The findings bridge personality psychology, group processes, and evolutionary perspectives, offering a framework to explore how personality diversity fosters collective intelligence.

## Introduction

Groups of living things often exhibit collective intelligence—the emergent group-level capacity to solve problems or make decisions effectively, often exceeding what individuals can achieve alone. While collective intelligence is a broad term typically used to describe intelligent group outcomes, the underlying group-level processes that generate such outcomes are often referred to as collective cognition, including how information is propagated and stabilized across members (in some cases, via artifacts and environmental representations; Hutchins, 1995) to produce agreement, deliberation, and closure. The current empirical study focuses on swarm intelligence—the emergent problem-solving capacity arising from decentralized agent interactions and information sharing that does not require centralized control or explicit global knowledge (Bonabeau et al., 1999; Kennedy and Eberhart, 1995)—as a mechanistic subset of collective cognition. Accordingly, we treat swarm intelligence as both (1) a process by which agent-level interactions can yield convergence and information integration, and (2) an outcome, quantified in this study as group-level performance across search landscapes.

While swarming behavior can emerge from agents following identical rules, effective intelligent swarming also requires behavioral diversity. In individual organisms, evolutionary theory emphasizes preserving core traits amid diverse expressions of others, providing ontogenetic and phylogenetic options via epigenetic expression and natural/sexual/kin selection. At the cellular level, bounded diversity is also evident. For instance, organisms survive only within specific temperature ranges and temperature reflects a Boltzmann distribution of energy states, where bounded randomness in molecular collisions drives cellular dynamic self-assembly. Analogous principles of bounded diversity appear across many physical scales, from molecular dynamics to quantum probability distributions. In the present research, we argue for the collective intelligence and cognition functionality of bounded personality diversity (i.e., some degree of personality diversity within a group will benefit that group) and propose a swarm intelligence computational method to test prediction derived from such arguments.

## A theory to test

Pringle and Robinson (2024) proposed bounded diversity advantage theory (BDAT) as an explanatory framework for personality traits, integrating personality psychology, evolutionary psychology (from a sociobiological and sociocultural dual-inheritance perspective), group process research, and the collective intelligence literature, while treating gene–culture coevolution as the primary evolutionary scaffold for claims about norms and group functioning. BDAT posits that bounded diversity in personality traits, such as the Big Five, drives information sharing and convergence dynamics (e.g., swarm intelligence processes) that improve collective performance (e.g., swarm intelligence outcomes). The present research, which uses agent-based swarm intelligence modeling, provides an initial computational test of BDAT derived predictions.

To clarify our assumptions when integrating BDAT with evolutionary psychology, our argument is compatible with an adaptationist perspective on psychological mechanisms (e.g., selection-shaped information-processing capacities), but it does not require strong commitments to any single disputed program (e.g., a strict “massive modularity” claim). BDAT’s group-level predictions are framed within dual-inheritance theory, such that stable psychological mechanisms interact with culturally transmitted norms and institutions. In this context, any claims about “selection” at the group level refer to cultural-evolutionary dynamics rather than genetic group selection.

### Bounded diversity advantage theory

Factor analysis of language led to the development of the Five Factor Model (FFM) of personality (Digman, 1990; Goldberg, 1993; McCrae and John, 1992) without a guiding theoretical framework. The FFM’s trait descriptors for human personality in WEIRD societies (Henrich et al., 2010)—along with the overlapping Big Five (Goldberg, 1990) and HEXACO (Ashton and Lee, 2020) models – have prompted post-hoc theoretical explanations for these “Big Few” traits, yet no consensus has emerged. Pringle and Robinson (2024) introduced BDAT, which interprets the Big Few through an evolutionary and group lens. BDAT argues that the sociocultural evolution of the FFM’s lexical descriptors selected for normative behaviors—and non-normative bounds—essential for effective collective intelligence, with “selection” here referring to differential cultural retention and transmission of norms that support group coordination. In this framework, diversity in personality across FFM dimensions, within normative bounds, fosters deliberation in the service of more optimal solutions for any group-based task, reducing group biases that cause premature closure around suboptimal solutions, thereby enhancing collective intelligence.

BDAT’s evolutionary account is intended as an integrative lens for linking personality structure to group function rather than a claim that any one evolutionary-psychology tradition uniquely explains the Big Five. Even so, BDAT extends two evolutionary theories of personality diversity—the Niche Diversity hypothesis and the Coral Reef model. The Niche Diversity hypothesis (Smaldino et al., 2019) argues that complex socioecological environments (e.g., urbanized societies with varied occupations) foster greater personality dimensionality, lower trait covariation, and higher variance by rewarding specialized trait combinations. The Coral Reef Model (Figueredo et al., 2005) posits that personality variation arises from social micro-niches in dense populations, driven by frequency-dependent selection (i.e., rare traits gain fitness advantages by allowing individuals to fill unoccupied roles, leading to character displacement and niche-splitting for reduced competition). BDAT serves as a bridge between the two models. All three models reject universal, fixed personality structures, instead viewing traits as adaptive responses to niche diversity for survival advantages—individual in Coral Reef, structural in Niche Diversity, and collective in BDAT, roughly speaking. This triad could inform unified models, outlining how historical social density (Coral Reef) leads to modern niche complexity (Niche Diversity), yielding group benefits (BDAT).

At the group level, BDAT serves as a swarm intelligence framework, grounded in gene-culture coevolution and cultural evolutionary accounts of norm formation. It posits that personality trait dimensions emerged from sociobiologically evolving (gene-driven) psychological mechanisms (e.g., Tooby and Cosmides, 2015) that cross-stabilized with socioculturally evolving (meme-driven) lexical descriptors of normative and non-normative behavior, where “meme-driven” is used as shorthand for culturally transmitted information and not a claim that cultural units replicate with gene-like fidelity. From this dual-inheritance perspective, while FFM trait dimensions may not be universal across cultures, their stability in WEIRD cultures is partly rooted in underlying mechanisms (e.g., Lukaszewski et al., 2020). These mechanisms evolved via natural/sexual/kin selection, prioritizing individual adaptiveness. In contrast, cultural evolution producing lexical descriptors along trait dimensions arose from selection pressures that tended to produce group benefits (Richerson et al., 2016), consistent with cultural group selection accounts of cooperation and norm enforcement.

As products of cultural evolution largely focused on the group—intertwined with psychological mechanisms largely focused on the individual—the Big Few, per Pringle and Robinson (2024), function as lexical descriptors in WEIRD cultures to describe and reinforce normative behaviors relevant to group functions. Within this framework, the Big Few provide a shared understanding for communicating essential dimensions of difference that shape group dynamics. These descriptors parallel cultural representations described by Durkheim (1965) and the dynamic social representations of Moscovici (2000).

From BDAT’s perspective, the FFM trait dimensions—extraversion, agreeableness, conscientiousness, neuroticism, and openness/intellect—each define functional behaviors within groups. Extraversion delineates normative ranges for interpersonal interaction and activity, termed “engagement” in BDAT. Agreeableness defines norms for group harmony, alignment, or agreement, labeled “closure.” Conscientiousness establishes norms for group “tasks” and resource use. Neuroticism sets norms for managing uncertainty or “risk” and openness/intellect defines norms regarding “change.” To perform these functions— engagement, closure, task, risk, and change—groups enforce norms via descriptive constructs for normative and non-normative behaviors along each dimension. Humans devote substantial unstructured time to gossip (Dunbar, 2004), which often takes the form of representing the personality of others (Kameda and Tindale, 2006), which in turn provides leverage points for influencing them (Boyd and Richerson, 1985). Using extraversion as an example, comments like “she can be a little shy” or “he’s very outgoing” describe normative extraversion levels, while “he’s like a stone” or “she’s a motormouth” highlight extraversion norm violations. Each FFM trait has descriptors that span from low to high levels, with extremes (low or high) risking group dysfunction.

Pringle and Robinson (2024) argue that personality research and parts of the group process tradition have often developed in parallel rather than in close dialogue (also see Parks, 2012; Steiner, 1972). This point should not be read to imply that the Big Few lack empirical links to teamwork processes and outcomes (for a recent review, see Jolić Marjanović et al., 2024). The potentially generative aspect of BDAT is therefore not the general idea that personality, diversity, and group performance are connected per se, but the more specific proposal that bounded trait diversity—canalized along FFM dimensions and bounded by norms via a dual-inheritance account—can reduce premature closure and, in turn, improve collective performance.

While BDAT is a framework for the Big Few personality traits, it is also a theory of collective intelligence and cognition that we examine using swarm intelligence modeling. To make this point and to generate relevant data, BDAT-derived predictions are tested using computational agent-based models of swarm intelligence in the context of a novel variant of Particle Swarm Optimization (PSO) applied to diverse mathematical search spaces. This PSO variant will be used to provide evidence for the benefits of normative diversity with respect to conscientiousness-like behavior while laying the groundwork for exploring other trait-related differences that can be similarly operationalized.

### Various group sizes

Groups of different sizes are discussed in different social science literatures and there is no dominant framework for distinguishing group sizes. In this paper, the labels micro, meso, and macro are used heuristically to distinguish levels of analysis rather than to impose fixed taxonomic cutoffs. Micro refers to individuals, dyads, and very small groups; macro refers to larger cultural populations; and meso refers to group sizes between micro and macro levels. The present study’s 50-agent swarm should therefore be read as an intermediate-sized computational collective used for modeling group-level coordination dynamics rather than as a literal face-to-face small team or as an attempt to adjudicate disputed size boundaries in the groups literature. BDAT is primarily concerned with how macro-level cultural-evolutionary processes may shape normative trait bounds that, when distributed across members, can influence the functioning of meso-level collectives.

### Complexity emerging from parsimonious models

Novel theoretical frameworks like BDAT, which propose explanations for complex behaviors, require hypothesis testing. BDAT asserts that human swarm intelligence emerges partly from bounded diversity along personality trait dimensions. Emergent phenomena (Baas and Emmeche, 1997) are consistent with complexity theory (Manson, 2001), often counterintuitively illustrating how intricate patterns can arise from simple agent interactions, as seen, for example, in bird flocks, economic systems, social networks, and snowflakes.

Traditional hypothesis testing occurs in laboratory or naturalistic settings, which are rife with uncontrolled sources of variability. BDAT also intends to capture complex group processes such as how language evolves and is utilized for norm enforcement from gossip (Dunbar, 2004) among group sizes up to Dunbar’s (1992) number (e.g., faculty members of a college). Obtaining data from enough larger groups to support statistical power at the group level would seem a daunting task. Simulation offers an alternative. Agent-based modeling (ABM) and other bottom-up methods simulate agents following parsimonious rules, revealing how local interactions can yield global complexity. To test frameworks like BDAT, we can apply such reductive strategies in a rigorous and controlled manner. Like all methods, ABM faces critiques, including whether modeled parsimony mirrors real-world processes. Despite dimensional gaps between simulations and reality, modeling is proving to be increasing generative and useful across sciences, with recent advances occurring in the social sciences (Smaldino, 2023).

Real-world behaviors, like group decision-making, involve numerous variables, many of which are unobservable, and testing requires *in situ* manipulation and analysis to separate effects from confounders. The complex behavior is observable, but capturing the underlying causal variables is difficult. In emergence-focused modeling, variable control is relatively straightforward because the environment is simulated, with confounders present only if intended to be present. The challenge is demonstrating emergence itself—that is, showing that complex behaviors or outcomes can arise from simple agent-level components, interactions, and environments aligned with the theoretical elements of the framework being tested.

Computational tools—models, algorithms, artificial intelligence, and data analytics—aid social sciences, as evident in the emerging field of computational social science (Conte et al., 2012; Lazer et al., 2009). Particle Swarm Optimization (PSO) is one ABM platform that would seem particularly suited to testing the BDAT focus on collective intelligence and cognition because PSO formalizes how decentralized information sharing can yield measurable collective performance. In the present work, a novel PSO variant was used to instantiate diverse and non-diverse conscientiousness-like behaviors in swarm compositions across a variety of search functions, with swarm intelligence (group-level performance outcomes) as the key focus. More broadly, the work explores whether PSO can serve as a generative modeling approach for psychology and the study of individual differences.

### Mathematically modeling swarm intelligence

Researchers have used mathematical models to simulate group behaviors and cognitive processes, with advances in computational power enhancing their scope. Of particular relevance, Reynolds (1987) introduced “boids,” bird-like agents following simple rules – avoiding collisions, aligning with neighbors, and steering toward center mass – to replicate flocking. Each boid starts with a random position and vector in a Cartesian system, adjusting based on nearby agents, yielding emergent, life-like motion from parsimonious algorithms. Reynolds (1987) work influenced computer graphics and swarm intelligence models, highlighting how complex behaviors emerge from simple rules that need not rely on extensive representational activity. These simulations naturally led to questions about the degree to which our own complex behavior, culture, and collective intelligence might be products of social interactions that follow simple rules (e.g., in the form of evolved psychological mechanisms).

Other modeling approaches include evolutionary computation, such as genetic algorithms (Holland, 1992), evolutionary programming (Fogel et al., 1996), evolution strategies (Rechenberg, 1984), and genetic programming (Koza, 1994). Related models have been developed to simulate social influence (discussed below), cultural evolution (Boyd and Richerson, 1985), memetics (Dawkins, 2016; Dennett, 1995), cultural algorithms (Reynolds, 1994; Richerson and Boyd, 2008), and adaptive culture model (Kennedy, 1998). In sum, there are many agent-modeling approaches that have been used and our review will concentrate on particle swarm optimization (PSO).

Some ABMs have incorporated personality and emotions (e.g., Jed et al., 2004; Jones et al., 2009; reviewed by Ahrndt et al., 2015), using typologies like Myers-Briggs (Campos et al., 2009; Myers and Myers, 2010) or the FFM (Durupinar et al., 2008). Many ABMs employ Belief-Desire-Intention (BDI) architecture (Rao and Georgeff, 1991), in which beliefs inform actions, desires motivate them, and intentions drive them. For studying personality’s role in culturally evolved swarm intelligence, PSO suits our needs (for an overview of non-PSO social ABMs, see Smaldino, 2023). Limited PSO applications to personality exist (Bentley and Lim, 2022; Lim & Bentley, 2018, 2019a, 2019b; Lim et al., 2023) and our approach differs from these prior personality-based instantiations of PSO, as will be detailed.

## Particle swarm optimization

Particle swarm optimization (PSO), developed by Kennedy and Eberhart (1995), is a leading swarm intelligence model, drawing from contemporaneous social influence models (e.g., Axelrod, 1997; Kennedy, 1998; Latané, 1996) and evolution-informed computational procedures (e.g., Fogel et al., 1996; Holland, 1992; Koza, 1994; Rechenberg, 1984). The foundational papers (Eberhart and Kennedy, 1995; Kennedy and Eberhart, 1995) have amassed over 111,000 citations, influencing fields beyond social psychology, such as computational science (reviews in Freitas et al., 2020; Shami et al., 2022; Wang et al., 2018).

When computationally modeling cooperative animal and human behavior, van den Bergh (2001) recommends the inclusion of five elements related to proximity (spatiotemporal calculations), quality (environmental responsiveness), diversity (avoiding narrow constraints), stability (ignoring minor changes), and adaptability (behavioral shifts). PSO incorporates all such elements (Wang et al., 2018). Originating in social psychology and engineering (Kennedy as psychologist, Eberhart as engineer), PSO finds optima in mathematical topologies (search functions) representing solutions, problems, or control surfaces. PSO has been generative, finding applications in neural network training, feature selection, power grid optimization, and domains like antenna design, biomedical engineering, networking, prediction, robotics, scheduling, security, and signal processing (Nayak et al., 2023).

In most PSO algorithms, agents, or particles, possess limited information. At each iteration, they make vector decisions within a search function based solely on distances to their current personal best (pBest), the swarm’s global best (gBest), and their current trajectory. They remain blind to the search function’s broader topology and retain no historical memory of the space beyond current bests. Despite this parsimony, swarms efficiently find global minima across various search functions. Full knowledge of the search space emerges collectively as an epiphenomenon produced by agent interactions and information sharing. This emergent swarm intelligence process makes PSO a good modeling approach to evaluate BDAT’s (Pringle and Robinson, 2024) assertion that the intelligence of group decision-making is significantly shaped by social information sharing, which is akin to what Pringle and Robinson (2024) referred to as “discourse,” while also allowing one to quantify collective performance.

### The PSO algorithm

In PSO, swarm agents search for a global optimum (where fitness is maximized or error is minimized) in a continuous search space or cost function, where each particle’s given location is a candidate solution for the search. Each agent searches the function via simple vector (velocity and position) equations, which update at time intervals called iterations or “ticks.” At tick zero, particles are randomly positioned. At each tick, each agent’s vector is influenced by its momentum (where it was heading and at what speed), the best location it has found thus far (called pBest, for personal best), and the best location that has so far been found by any agent in the swarm (called gBest, for global or group best). Additional agent behavior is impacted by tuning or controlling coefficients that determine the weighting of influence of pBest (also called the “cognitive” coefficient c_1_) and gBest (also called the “social” coefficient c_2_), as well as randomness (r_1_ and r_2_) that creates bounded diversity in these weightings across the swarm. These acceleration coefficients tune performance, balancing exploitation (c_1_-dominant, personal knowledge) and exploration (c_2_-dominant, group knowledge), akin to exploit/explore tradeoffs (Cohen et al., 2007). Early performance tends to favor broad exploration, which, at some point, shifts to focused exploitation. Tuning c_1_ and c_2_ optimizes for specific applications, whereas defaults like c_1_ = c_2_ = 1.49445 suit broad search spaces (McCaffrey, 2011).

#### PSO equations, momentum, and inertia

Equations 1, 2 constrain change in velocity and position for each agent at each iteration.


$$\begin{array}{l} v_{\mathrm{id}}(t+1)=v_{\mathrm{id}}(t)+r_{1}c_{1}(\text{pBes}t_{\mathrm{id}}(t)-x_{\mathrm{id}}(t))\\ +r_{2}c_{2}(\text{gBes}t_{d}(t)-x_{\mathrm{id}}(t))\; \end{array}$$ (1)

$$\;\;x_{\mathrm{id}}(t+1)=x_{\mathrm{id}}(t)+v_{\mathrm{id}}(t+1)\;\;$$ (2)

**Figure 1:**
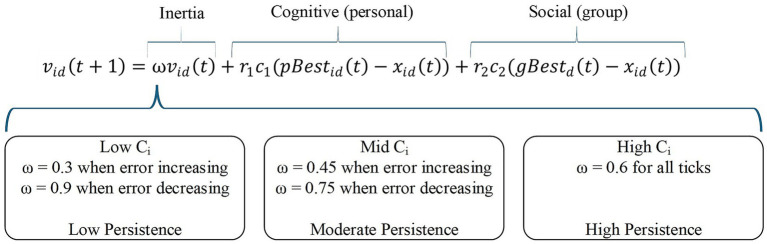
Vector influences on agents from pBest and gBest. This graphic shows how pBest and gBest (scaled by r_1_c_1_ and r_2_c_2_) influence the velocity vector of an agent, steering it to a new position.

**Figure 2:**
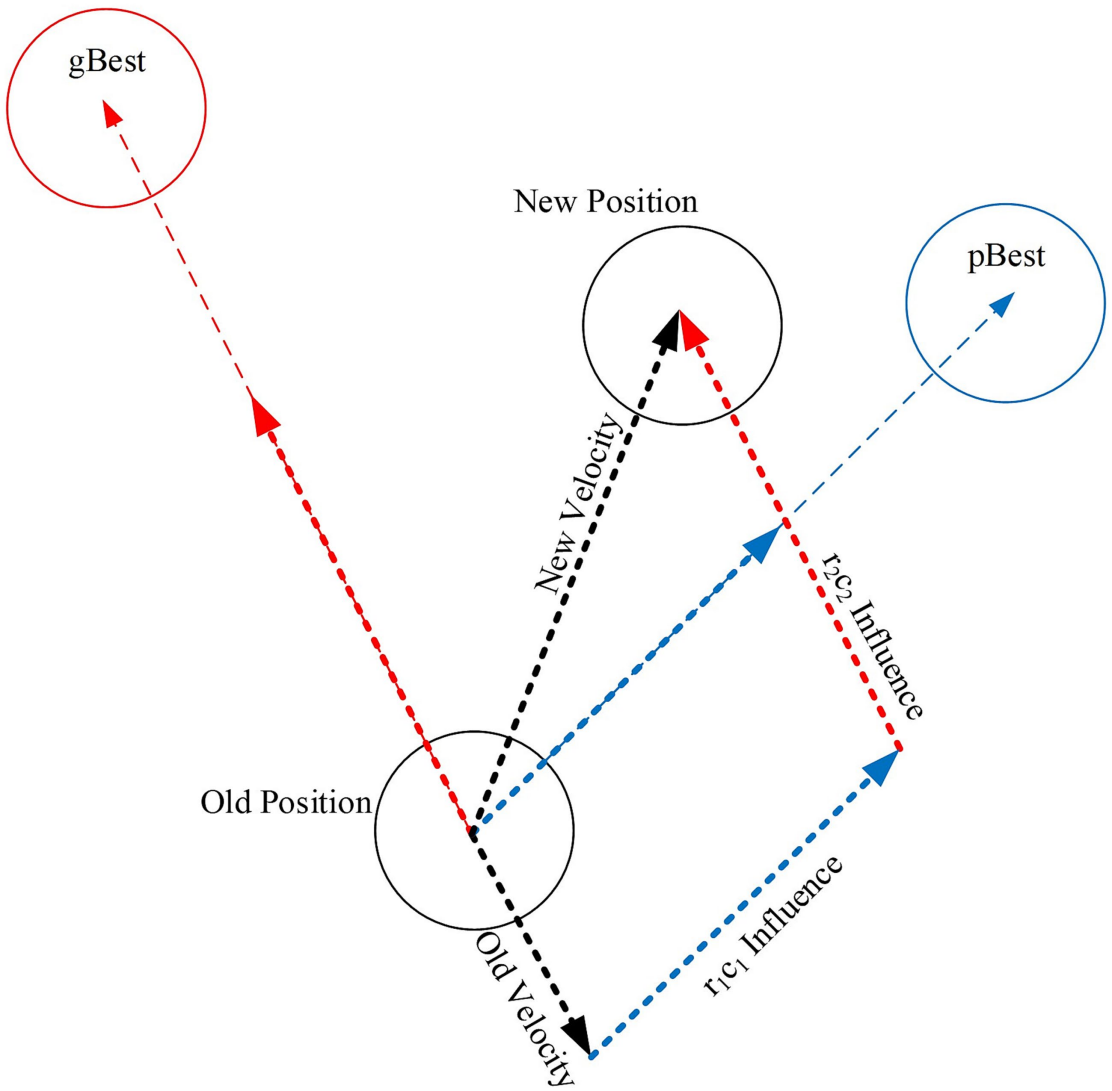
Flowchart of canonical PSO algorithm. This flowchart describes the basic algorithm of canonical particle swarm optimization (PSO). Many aspects of a computer program needed to perform PSO are not shown.

Here, r_1_ and r_2_ are uniform random [0,1] for each agent. Momentum (*v_id_*(*t*)) dampens abrupt changes and counters deceleration near pBest/gBest. Distance to pBest/gBest scales influence, resulting in agents decelerating as they get closer to pBest/gBest, mitigating overshooting. If c_1_ > c_2_, exploitation is biased, which is sometimes better for multimodal surfaces; if c_2_ > c_1_, exploration is biased, which is sometimes better for unimodal surfaces. Figure 1 illustrates how agents update their location at each tick and Figure 2 shows the flowchart of the canonical PSO algorithm.

Shi and Eberhart (1998) added inertia – signified by *ω* – to modulate momentum, which is now standard. Some PSO variants decelerate (across ticks) from 0.9 to 0.4 inertia (Eberhart and Shi, 2000; Xin et al., 2009), which enables early fast exploration to transition to fine exploitation. Other variants of inertia include random (Eberhart and Shi, 2000), nonlinear (Jiao et al., 2008), or alternatives like constriction (Clerc and Kennedy, 2002). For a listing of inertia approaches, see Kassoul et al. (2021). Our novel PSO variants will use the inertia parameter to operationalize conscientiousness-like behavior.

#### Programming PSO models

PSO requires more than just equations. Other functions must be instantiated in the program depending on the desired PSO variant and the intent of the study, including initializing random positions and r_1_/r_2_ values for each agent, tracking/updating pBest/gBest and running the algorithm equations through the search function equation, capping velocity (Vmax) to prevent overshoot, setting particle count, determining max iterations, and running simulations for establishing trial-level performance. Features must be added for user inputs, which typically define dimensions, bounds, functions, the number of simulations per trial, and other features that are desired to be user-determined. Libraries like NetLogo (Tisue and Wilensky, 2004) and PySwarms (Miranda, 2018) can facilitate these efforts. The current study used PySwarms libraries stitched with Python code to instantiate a novel set of PSO variants.

### Search functions for PSO

PSO searches mathematically defined spaces with fitness/error values at each point. Industrial uses often involve many dimensions, as do many benchmarks functions to test PSO variants (e.g., Abed et al., 2021; Andrei, 2008; Molga and Smutnicki, 2005; Test Function, 2024). The current study is limited to 2D functions plus an error dimension (i.e., error forms a z-axis surface with global minimum as the target), which accords with spatial navigation among groups (e.g., in finding habitable areas or hunting), and spatial metaphors can also be used to model other group-based decisions, such as deciding on the best solution to a problem in resource allocation. PySwarms, which was the library used in the present research, includes the benchmark test functions of Ackley, Beale, Booth, Bulkin’s No. 6, Cross-in-Tray, Easom, Eggholder, Goldstein, Himmelblau, Holder Table, Levi, Matyas, Rastrigin, Rosenbrock, Schaffer No. 2, Sphere, and ThreeHumpCamel.

#### Search functions as models for human decision-making

Search functions, which are models for situations, vary along continua such as convex/unimodal versus non-convex/multimodal, smooth versus rugged, and simple versus complex/nonlinear. Such function differences can be thought of in terms of different types of group decisions (e.g., unimodal when there is one correct answer, multimodal when there are tradeoffs, convex when iterative improvements can occur, and non-convex when the group needs to engage in strategic exploration). PySwarms’ diversity of available functions suffices with respect to these different sorts of problems or group-decisions spaces.

To make spatial metaphors more concrete without implying one-to-one real-world equivalence, consider three illustrative examples. Ackley is a rugged, multimodal landscape with many attractive local basins. It resembles search problems that might have a solution landscape akin to early-stage drug discovery or materials design, where many candidate solutions look “promising enough” locally but only a small region yields a truly safe/effective compound or configuration. Rosenbrock’s narrow, curved valley (“banana” structure) might resemble coordination problems in which progress requires a sequence of mutually compatible small moves, akin to multi-stakeholder negotiation or policy compromises where many steps can feel locally sensible yet only a carefully coordinated path yields improvement. By contrast, Sphere is a smooth, convex bowl more akin to well-posed problems where gradient-like improvements are consistently informative and rapid closure is typically beneficial. These vignettes are intended to help readers appreciate the point that different search landscapes pose different risks of premature closure and why bounded behavioral diversity may matter most when the environment contains misleading local optima.

According to Surowiecki (2005), groups make decisions that involve cognition (e.g., reasoning), coordination (e.g., planning), and cooperation (e.g., by assigning roles) across topologies representing plausible hypotheses or possible solutions landscapes. Such topological metaphors trace to Waddington (1957) and Lewin (2015), though they can become literal when spatial navigation is involved. BDAT (Pringle and Robinson, 2024) contends that personality diversity prevents premature closure (Kruglanski et al., 2006), disrupting settling on local minima via discourse. Agents with prosocial traits tend to favor early group consensus whereas agents with assertive, open, or antagonistic traits extend deliberation. Groups typically work on problems that lack obvious solutions, rendering deliberation useful.

From the present perspective, then, diverse search functions can be conceptualized in terms of diverse group decision situations. Applying personality-like behavior to PSO agents with respect to only a single search function runs the risk of making conclusions that do not generalize to other search spaces. We sought to avoid this problem by comparing groups defined by personality variation across multiple search functions that differ in many respects.

## Agent variations in conscientiousness-like behavior

In the present research, we created agents that could be described in terms of the conscientiousness dimension of personality. This focus is intriguing because BDAT proposes that the presence of some “lazy” group members can increase swarm intelligence. Unlike extraversion or agreeableness, too, conscientiousness is primarily thought of in terms of self-regulation rather than social functions. Nonetheless, we aim to show that this dimension of personality is relevant to group decision-making, with the idea that groups with diverse conscientiousness levels will tend to perform better than more homogenous groups.

### Conscientiousness: a brief review

Conscientiousness is a core FFM trait (Goldberg, 1993) that is conceptually rooted in Freud’s ideas concerning the id’s use of the pleasure principle and the ego’s role in impulse control (Roberts et al., 2014). Pre-FFM, conscientiousness had guises such as ego control (Block and Block, 2014) or constraint versus disinhibition (John et al., 2008). Related constructs also include delay of gratification, effortful control, self-regulation, and grit, all of which describe tensions between long-term goal pursuit and behavior that is responsive to more momentary impulses (Baumeister and Heatherton, 1996).

Roberts et al. (2009) define conscientiousness in terms of self-control, responsibility, hard work, organization, and rule adherence. Facets include orderliness, industriousness, self-control, responsibility, traditionality, decisiveness, formality, punctuality, persistence, and virtue (Roberts et al., 2014). DeYoung et al. (2007) split conscientiousness into two aspects named orderliness (planning, organization, tradition, cleanliness) and industriousness (hard work, achievement, persistence). Persistence links to industriousness (De Raad and Peabody, 2005; MacCann et al., 2009) and grit (Duckworth et al., 2007), which are also encompassed by conscientiousness (Rimfeld et al., 2016). BDAT (Pringle and Robinson, 2024) links conscientiousness to group functions related to task and resource use, with higher levels of conscientiousness supporting greater investment (i.e., working hard on the problem at hand). Although organizational and cultural norms tend to favor higher levels of conscientiousness, BDAT challenges this “higher is better” framing from a group decision-making perspective.

### Operationalizing conscientiousness-like behavior in PSO

Comprehensively mapping a broad trait like conscientiousness onto a single agent-based modeling (ABM) algorithm is neither achievable nor desirable. It is also true that BDAT (Pringle and Robinson, 2024) recognizes that not all facets of conscientiousness are relevant to group decision-making. Distinguishing facets driven by group-level cultural evolution and selection from those rooted in individual psychological mechanisms will require an extensive research program. However, the distinction between the aspects of organization and industriousness (DeYoung et al., 2007) appears to be a useful one. Industriousness, encompassing up to half of conscientiousness facets (DeYoung et al., 2007), can be modeled in PSO, as will be described, with persistence as the most particular way of thinking about what can be operationalized.

## Method

### Operationalization of core constructs

Although Pringle and Robinson (2024) did not anticipate PSO as a means of testing BDAT, we now see that BDAT’s theoretical elements can map naturally onto swarm intelligence agent-based modeling, albeit in a reduced manner, which is both intentional and desirable from an ABM perspective. In line with ABM standards, we emphasize clear mechanism-to-measure links and carefully bounded conclusions rather than the richer realism of communication and role structure that is often expected in small-group/team modeling traditions. We treat collective intelligence as an outcome construct and operationalize it using a novel performance measure that captures the speed–accuracy efficiency of group problem solving across search environments. We also treat collective cognition as a process construct and operationalize it using convergence-threshold dynamics that index how rapidly and reliably a group stabilizes on candidate solutions (e.g., majority, consensus, and unanimity thresholds). Within this framework, PSO instantiates swarm intelligence in terms of decentralized local updates and information sharing that generate group-level coordination without centralized control or explicit global knowledge (Bonabeau et al., 1999; Kennedy and Eberhart, 1995). In our model, conscientiousness-like industriousness/persistence is operationalized as variation that is constrained within a permitted range, which is a proxy for normative bounds. In this way, BDAT’s norm canalization is an environmental constraint that scaffolds in a distributed cognition sense (Hutchins, 1995) to influence both collective cognition and intelligence.

It should also be stated that BDAT is a broad framework that is not fully reducible to swarming. On this point, the current contribution is particular to the subset of BDAT mechanisms that can be instantiated as decentralized interaction, bounded behavioral variation, and emergent stabilization on candidate solutions. Our inferences are limited to these swarm-like components of BDAT, though the research will show that BDAT lends itself to controlled testing and the research does focus on a core BDAT prediction, namely that bounded trait diversity can mitigate premature closure and improve group-level performance.

We recognize that the current research represents the first empirical test of BDAT and that it relies on a novel computational operationalization. Accordingly, this study does not test BDAT in isolation but evaluates whether a computational instantiation produces theory-consistent patterns; any support should therefore be interpreted as conditional on this operationalization rather than as a definitive validation of the full theory. With such caveats acknowledged, the results would support BDAT-based theorizing if two central predictions received support. First, compositions with bounded diversity in conscientiousness-like behavior were predicted to outperform non-diverse compositions across search environments and agreement thresholds. Second, within the polarized condition, high-C_i_ agents should drive early convergence whereas low-C_i_ agents should prolong exploration. Failure to observe these patterns would lead to some reconsideration of BDAT or the present operationalizations.

### Model setup and operationalizing conscientiousness

In our PSO variant, high-industriousness agents (denoted as high-C_i_) exhibit strong steady inertia, maintaining their trajectory despite obstacles and short-term deviations from progress indicators in their environment. The relevant tendencies are reflected in persistent, effort-intensive behavior akin to “grinding away” or “staying on course” (Roberts et al., 2014). Conversely, low-industriousness agents (low-C_i_), in the instantiated model, display less persistence, reducing effort when obstacles are encountered and energetically moving when the going is easy. Both such trends operationalize a sensitivity to short-term costs and benefits that is often viewed as a key to how self-regulation varies as a function of conscientiousness (Baumeister and Heatherton, 1996; Roberts et al., 2014).

**Figure 3:**
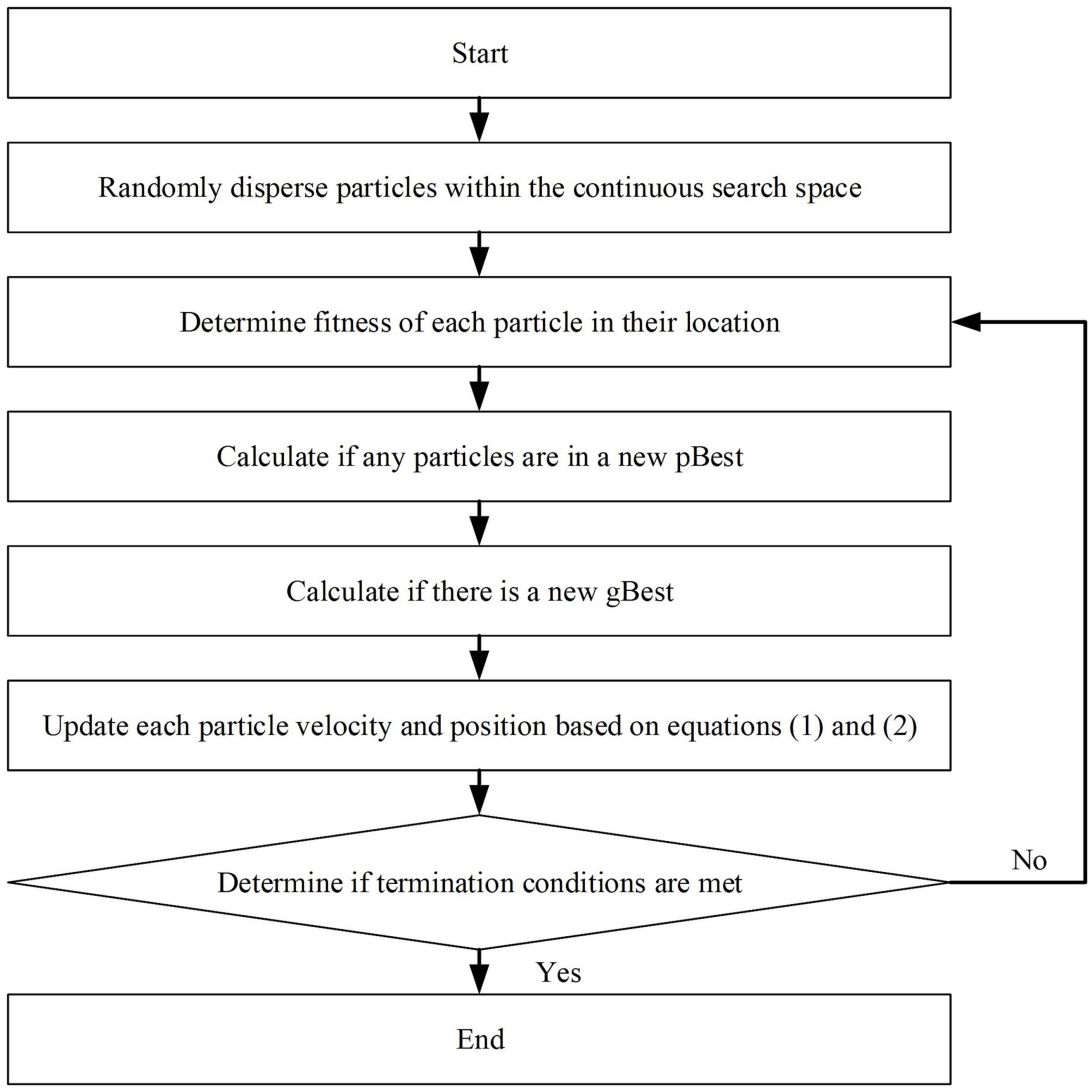
Operationalization of conscientiousness via industriousness/persistence as adaptive inertia. This diagram shows changes to the inertia parameter that impart industriousness or persistence in PSO swarm agents.

We implemented our variant of PSO using Python 3.12, PyCharm Community Edition 2024.2, and the PySwarms libraries. In BDAT, variations in conscientiousness reflect variations in task persistence, particularly when progress is slow or non-evident. Under such conditions, high conscientiousness (high-C_i_) agents should persist and low conscientious agents should lessen their efforts, reflecting sensitivity to momentary incentives and costs (Baumeister and Heatherton, 1996). Operationally, and using a baseline inertia of *ω* = 0.6, high-C_i_ agents were those that maintained ω = 0.6 regardless of direction, embodying steady effort. Mid-C_i_ agents adjusted by ±0.15 (ω = 0.75 downhill, 0.45 uphill) and low-C_i_ agents adjusted by ±0.3 (ω = 0.9 downhill, 0.3 uphill), with this operationalization shown in Figure 3.

BDAT contends that bounded personality diversity will benefit collective intelligence. In our PSO approach, this key prediction will be examined by comparing the performance of six group compositions: High (all high-C_i_), Mid (all mid-C_i_), Low (all low-C_i_), Polar (50% high-C_i_, 50% low-C_i_), Mixed (equal high-, mid-, low-C_i_), and Mixed-U (inverted U-shaped: 26% high-C_i_, 48% mid-C_i_, 26% low-C_i_). Diversity of conscientiousness-like behavior is high in the Mixed and Mixed-U composition conditions, medium in the Polar condition, and low in the High, Mid, and Low composition conditions, with the latter three conditions varying in average levels of conscientiousness for the sake of studying how conscientiousness manifests itself irrespective of diversity considerations.

### Search functions and simulation parameters

Swarms comprised 50 agents navigating 2-dimensional mathematical search spaces, with positions and velocities updated per Equations 1, 2. Adaptive inertia was used to modulate momentum, with acceleration coefficients c_1_ = c_2_ at 1.49445, where r_1_ and r_2_ were uniform random [0,1], and the velocity clamp was set to 0.8. Search bounds were standard from the libraries for each function and any agents exceeding search bounds re-entered from the opposite side. All parameters and procedures were standard, then, with the exception that momentum was used to create agents varying in conscientiousness-like behavior.

Additional innovations occurred in defining group performance. In this connection, we used the behavior of the swarm to define swarm “agreement,” defined in terms of the number of agents eventually clustering within a radius of the current gBest solution for a given function, as an operational index of collective adoption/closure rather than merely the best individual location. Various convergence levels were included, from majority (i.e., 25 of the 50 agents clustered within the convergence zone) to unanimity (i.e., all 50 agents clustered within the convergence zone). Convergence rates were acceptable for 11 of the 17 available PySwarms benchmark functions: Ackley (99.83%), Beale (96.83%), Booth (99.83%), Goldstein (100%), Levi (100%), Matyas (100%), Rastrigin (100%), Rosenbrock (98.67%), Schaffer No. 2 (99.83%), Sphere (99.83%), and Three-Hump-Camel (99.83%). The percentage of simulations resulting in 100% convergence (i.e., unanimous agreement of the swarm) within 2000 iterations was too low for the functions of Bukin No. 6 (1.50%), Cross-in-Tray (77.83%), Easom (77.00%), EggHolder (0%), Himmelblau (1.33%), and HolderTable (0%). Results will therefore be based on the 11 functions that supported sufficient convergence rates, with Table 1 characterizing each function in terms of bounds and other relevant characteristics.

**Table 1:** Characteristics and bounds of the 11 search functions used in PSO simulations.

Function	Characteristics	Bounds (each d)
Ackley	Non-Convex, Multimodal, One Global, Smooth, Complex, Conical	-5 to 5
Beale	Non-Convex, Multimodal, One Global, Smooth, Complex, Flat	−4.5 to 4.5
Booth	Convex, Unimodal, One Global, Smooth, Non-Complex, Flat	−10 to 10
Goldstein	Non-Convex, Multimodal, One Global, Smooth, Complex, Flat	−2 to 2
Levi	Non-Convex, Multimodal, One Global, Smooth, Complex, Conical	−10 to 10
Matyas	Convex, Unimodal, One Global, Smooth, Non-complex, Flat	−10 to 10
Rastrigin	Non-Convex, Multimodal, One Global, Smooth, Complex, Conical	−5 to 5
Rosenbrock	Non-Convex, Unimodal, One Global, Smooth, Complex, Flat	−5 to 5
Schaffer No. 2	Non-Convex, Multimodal, One Global, Smooth, Complex, Conical	−100 to 100
Sphere	Convex, Unimodal, One Global, Smooth, Non-complex, Flat	−100 to 100
3HumpCamel	Non-Convex, Multimodal, One Global, Smooth, Complex, Flat	−5 to 5

This table summarizes the 11 two-dimensional search functions, their topological characteristics (e.g., convexity, modality), and dimensional bounds applied in the PSO simulations.

We ran 100 simulations for each combination of group composition (6) and function (11), with a limit of 2000 iterations (ticks) per simulation. Pre- and post-study exploration involving higher and lower agent populations, numbers of simulations per composition/function combination, and ticks, as well as small changes to c1 = c2 and the velocity clamp, did not affect conclusions. Conclusions are therefore based on 100 simulations per composition/function combination in the context of 2000 allowable iterations.

### Performance measurement

Beyond simply tracking the global best error (gBest), which is typically the only metric of most PSO applications, we also monitored swarm agent spatial convergence around gBest as a proxy for group agreement, thereby distinguishing the best-found solution from the degree to which the collective adopted it. With respect to this methodological innovation, the convergence zone (a radius distance = 0.01 from gBest) defined “agreement” based on the portion of the swarm within the zone. This convergence zone radius was determined empirically as the practical trade-off of time (number of ticks to reach an agreement threshold) and accuracy (spatial proximity to the gBest location). Exploration of larger (0.1) and smaller (0.001) zone radii did not alter conclusions and the radius was therefore set to 0.01. Convergence thresholds (percent of swarm within the zone radius) were set at 50% (representing a majority agreement), 66% (consensus), 90% (strong consensus), and 100% (unanimity). For each simulation, we recorded gBest error for a given threshold and the number of ticks to reach that threshold.

Error values were non-commensurate across functions, with overall averages ranging from 0.0000000000000884729 (Schaffer No. 2) to 3.000004071 (Goldstein). To render these numbers comparable, we z-scored across compositions for each combination of threshold and function, with raw numbers representing averages across 100 simulations. We also computed tick-based z-scores in a similar manner. Finally, we created a novel Performance Efficiency Score (PES) by averaging z-scores for ticks and errors, with lower numbers indicating that the particular composition exhibited better performance, defined as being faster with less error. At this point, the performance of different composition groups could be directly compared. Swarm intelligences is therefore defined in terms of collective problem-solving performance captured by a speed–accuracy–agreement triad involving solution quality (error), time cost (ticks), and explicit agreement thresholds (the proportion of agents that have converged). Our novel performance metric, PES, summarizes a group’s performance under a speed–accuracy tradeoff for each agreement threshold across functions. PES can also be collapsed across agreement thresholds to produce an overall performance summary.

According to BDAT, finally, low and high conscientious individuals will tend to serve different functions in a group. High conscientious individuals will tend to close on a solution, whereas low conscientious individuals will often extend group deliberation, which can be useful under many circumstances (Kruglanski et al., 2006). In PSO, these distinct functions will tend to take the form of high-C_i_ agents converging more quickly, with low-C_i_ agents continuing to explore (meander) away from the convergence zone. To examine such predictions, we counted the number of high- and low-C_i_ agents that were in the convergence zone at the 50% threshold for the polar composition, which consists of an equal number of high- and low-C_i_ agents. These numbers were computed at the simulation level, but averages will be compared.

### Translations of group processes and group performance

To reiterate, the present model uses a 50-agent computational collective to examine group-level coordination and convergence dynamics. We do not treat this swarm as a literal small team, but as a tractable approach to examine whether bounded behavioral diversity can influence collective search and closure. Swarm-related decision-making is defined in terms of movement toward and adoption of gBest via convergences. The percentage of agents within the defined radius around gBest is a proxy for group agreement. Deliberation is the phase of the trial that occurs prior to convergence. What might be thought of in terms of discourse involves the integration of information related to pBest and gBest. Such translations relate PSO parameters to group decision-making.

### Data analysis

Performance parameters were z-scored by threshold and function, rendering an ANOVA-based platform problematic, and it was also the case that we wished to focus on means across simulations rather than dropping down to the simulation level. Some of our inferential analyses therefore used a nonparametric sign-test approach that took advantage of 44 function-by-threshold observations for each composition. Specifically, we counted the number of cases in which one composition (e.g., Mixed) exhibited directionally superior performance relative to another composition (e.g., High) and evaluated such differences using sign tests. This analysis strategy will be supplemented by other strategies, both descriptive and inferential, for characterizing trends in the results.

## Results

**Figure 4:**
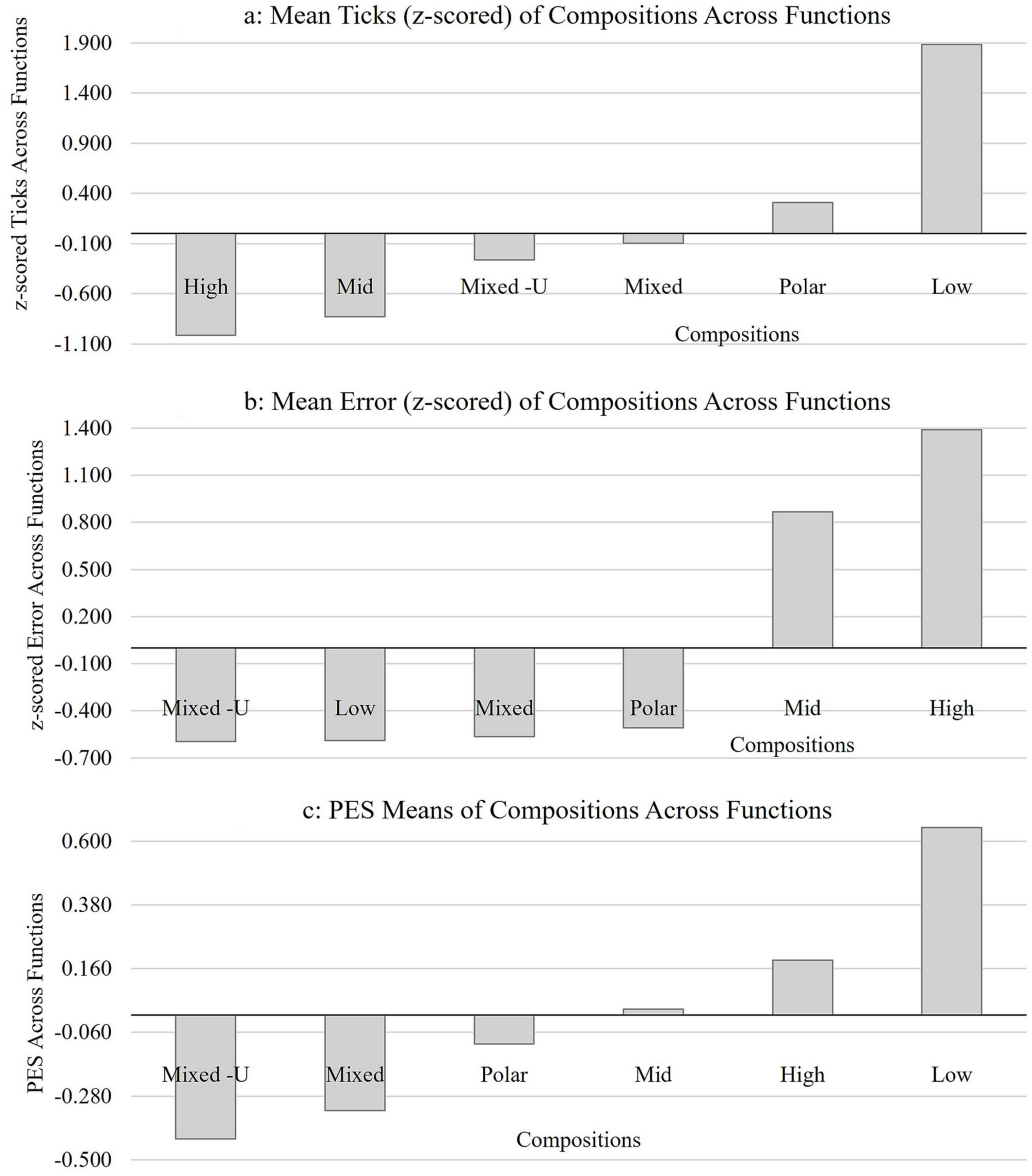
Means across all convergence thresholds and test functions for ticks, error, and PES.

Bounded Diversity Advantage Theory (BDAT) predicts that groups with diverse levels of conscientiousness will perform better than non-diverse groups. This prediction can be first examined in relation to PES means, reported in Table 2 and Figure 4c, that have been collapsed across thresholds and functions. As exhibited in Table 2 and Figure 4c, there was strong support for hypotheses, with mixed groups (Mixed-U, Mixed, and Polar) displaying negative z-scores and non-mixed groups (Low, Mid, and High) exhibiting positive z-scores.

**Table 2:** Mean ticks, error, and PES of group performance by composition across functions.

Group	Ticks	Error	PES
Mixed -U	−0.26	−0.59	−0.43
Mixed	−0.09	−0.56	−0.33
Polar	0.31	−0.51	−0.10
Mid	−0.83	0.87	0.02
High	−1.01	1.39	0.19
Low	1.88	−0.59	0.65

Means are collapsed across 4 agreement thresholds and 11 search functions.

PES combines considerations related to speed (ticks) and accuracy (error), but BDAT also makes predictions concerning these separable components of performance. Specifically, BDAT contends that high-C_i_ groups will tend to converge somewhat quickly (lower ticks), but with higher error, in part due to convergence speed. By contrast, low-C_i_ groups will converge slowly (higher ticks), but in the context of relatively lesser error, in part due to greater deliberation. These predictions can also be examined with respect to means that are collapsed across compositions and thresholds, with these means displayed in Table 2, Figure 4a (ticks), and Figure 4b (error). Consistent with predictions, the Low group was routinely the slowest to converge and the High group exhibited the greatest error.

Inferential testing took advantage of the fact that performance could be defined with respect to 11 search functions and 4 thresholds—i.e., 44 relevant data points. For each performance metric (ticks, error, PES) separately, we counted the number of times (out of 44) than one group performed better than another group, defined in terms of a directionally lower z-score. Because there were 6 compositions, there were also 15 pairwise composition comparisons. These analyses take advantage of aggregation, with each value reflecting 100 simulations, and the number of opportunities to compare means (44). They also give equal weighting to each comparison, ensuring that results generalize across search functions.

Results for these comparisons are reported in Table 3, which details the number of times that one group performed better than another (out of 44) and tests the significance of the relevant comparison in terms of a sign test. If one group outperformed another 29 out of 44 times, the relevant frequencies would be different from each other, as indicated by a χ^2^ value and its associated *p* value. These analyses were highly informative because, in the vast majority of cases (15/15 for ticks, 14/15 for error, 14/15 for PES), one group did outperform another group. In all cases of significance, furthermore, the direction favored the composition that BDAT would favor. For example, with respect to PES values, the Mixed-U group performed better than all other groups, the mixed group performed better than all other groups aside from the Mixed-U group, and so forth. The pattern of group differences will be more fully explored below.

**Table 3:** Pairwise contrasts of mean ticks, error, and PES for 44 combinations of convergence thresholds and test functions.

Group	Ticks	Error	PES
	Contrast, Chi-Square, p-value	Contrast, Chi-Square, p-value	Contrast, Chi-Square, p-value
Mixed-U/Mixed	42/2, χ^2^ = 35.56, *p* < 0.001	14/30, χ^2^ = 5.82, *p* < 0.016	39/5, χ^2^ = 26.27, *p* < 0.001
Mixed-U/Polar	42/2, χ^2^ = 35.56, *p* < 0.001	9/31, χ^2^ = 7.36, *p* < 0.001	38/6, χ^2^ = 23.27, *p* < 0.001
Mixed-U/Mid	4/40, χ^2^ = 29.45, *p* < 0.001	44/0, χ^2^ = 44, *p* < 0.001	34/10, χ^2^ = 13.09, *p* < 0.001
Mixed-U/Low	44/0, χ^2^ = 44, *p* < 0.001	25/19, χ^2^ = 0.82, *p* < 0.366	44/0, χ^2^ = 44, *p* < 0.001
Mixed-U/High	0/44, χ^2^ = 44, p < 0.001	44/0, χ^2^ = 44, p < 0.001	35/9, χ^2^ = 15.36, *p* < 0.001
Mixed/Polar	42/2, χ^2^ = 35.56, *p* < 0.001	11/33, χ^2^ = 11, *p* < 0.001	39/5, χ^2^ = 26.27, *p* < 0.001
Mixed/Mid	3/41, χ^2^ = 32.81, *p* < 0.001	43/1, χ^2^ = 40.09, *p* < 0.001	34/10, χ^2^ = 13.09, *p* < 0.001
Mixed/Low	44/0, χ^2^ = 44, *p* < 0.001	15/29, χ^2^ = 4.45, *p* < 0.035	44/0, χ^2^ = 44, *p* < 0.001
Mixed/High	0/44, χ^2^ = 44, *p* < 0.001	43/1, χ^2^ = 40.09, *p* < 0.001	34/10, χ^2^ = 13.09, *p* < 0.001
Polar/Mid	2/42, χ^2^ = 35.56, *p* < 0.001	42/2, χ^2^ = 35.56, *p* < 0.001	28/16, χ^2^ = 3.27, *p* < 0.070
Polar/Low	44/0, χ^2^ = 44, *p* < 0.001	33/11, χ^2^ = 11, *p* < 0.001	42/2, χ^2^ = 35.56, *p* < 0.001
Polar/High	0/44, χ^2^ = 44, *p* < 0.001	42/2, χ^2^ = 35.56, *p* < 0.001	32/12, χ^2^ = 9.09, *p* < 0.003
Mid/Low	44/0, χ^2^ = 44, *p* < 0.001	0/44, χ^2^ = 44, *p* < 0.001	40/4, χ^2^ = 29.45, *p* < 0.001
Mid/High	1/43, χ^2^ = 40.09, *p* < 0.001	31/13, χ^2^ = 7.36, *p* < 0.007	30/14, χ^2^ = 5.82, *p* < 0.016
Low/High	0/44, χ^2^ = 44, *p* < 0.001	44/0, χ^2^ = 44, *p* < 0.001	1/43, χ^2^ = 40.09, *p* < 0.001

Counts reflect the number of times that one composition outperformed another composition across thresholds and functions.

**Figure 5:**
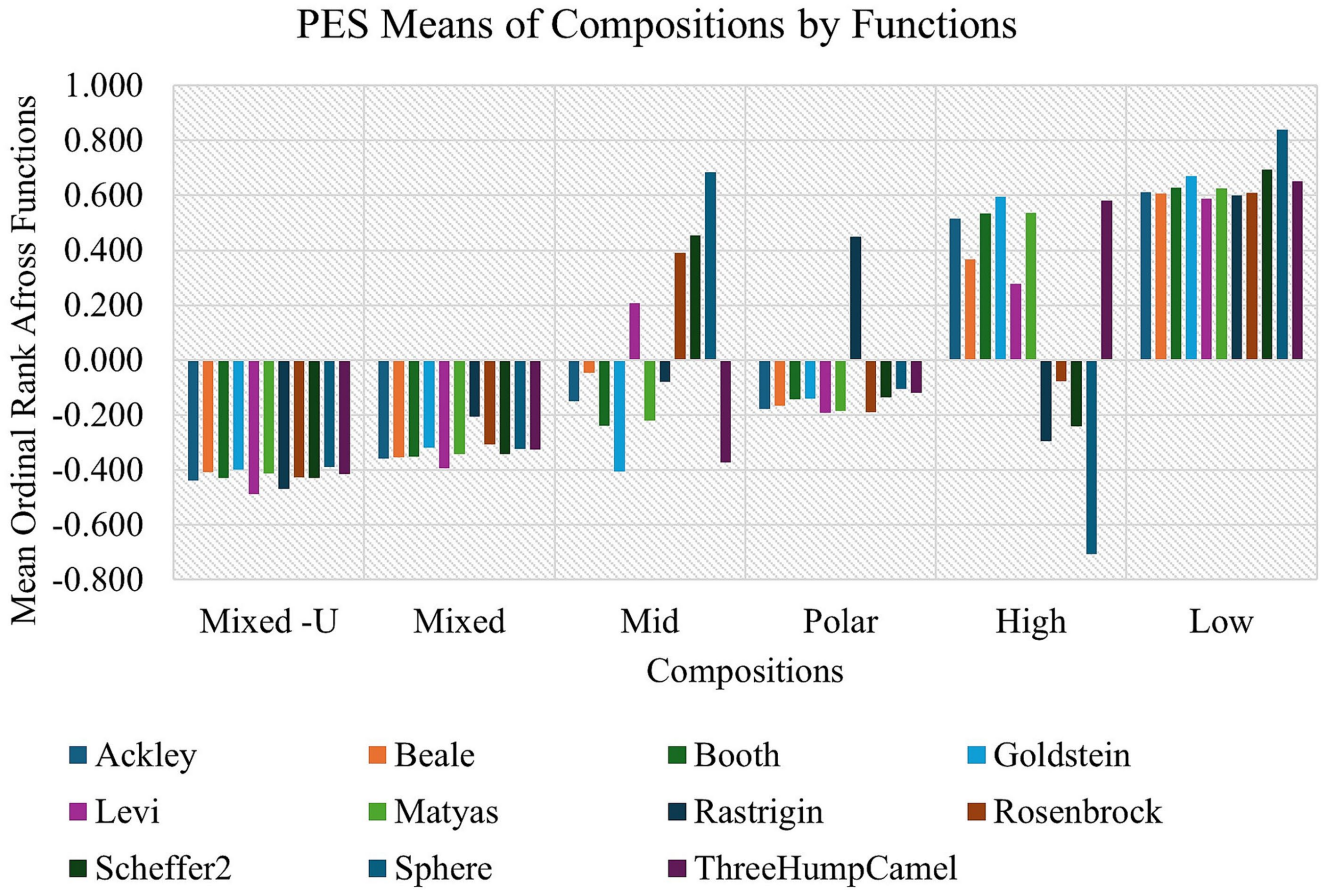
Mean PES for compositions across all 4 convergence thresholds for each search function.

Inferential statistics could not be used for particular test function environments, but, for the sake of interest, Figure 5 graphs PES values by composition and function, collapsing across convergence thresholds. Figure 5 complements Table 3 by indicating that the predicted results are not particular to some search functions, but rather the pattern of group comparisons cuts broadly across functions, though this point will be revisited in the Discussion section. BDAT favors mixed groups and mixed groups (Mixed and Mixed-U) performed consistently well across environments. The low-C_i_ group consistently performed poorly. The performance of the remaining groups varied by environment. In another analysis, it was found that group composition differences tended to retain their pattern across thresholds, with these cross-threshold correlations reported in Table 4.

**Table 4:** Correlation of performance across convergence thresholds.

Threshold	Ticks to Ticks			Error to Error			PES to PES		
	50%	66%	90%	50%	66%	90%	50%	66%	90%
66%	0.97	—		0.96	—		0.95	—	
90%	0.85	0.93	—	0.80	0.82	—	0.71	0.75	—
100%	0.71	0.81	0.97	0.81	0.79	0.91	0.53	0.55	0.80

The correlations indicate that compositions that perform better at one threshold perform better at other thresholds as well.

All 6 groups can be vectorized in terms of the number of low-, mid-, and high-C_i_ agents. For example, the number of high-C_i_ agents is 0 for the Mid condition, 13 for the Mixed-U condition, 25 for the Polar condition, and 50 for the High condition. These counts were correlated with means for tick, error, and PES z-scores that were averaged across threshold and environment, with these results being reported in Table 5. The number of low-C_i_ agents for a particular composition shared a very strong relationship with convergence speed, which was much slower if there were more low-C_i_ agents. This vector was negatively related to error, however, resulting in a PES-based correlation of 0.52. More mid-C_i_ agents was linked to faster convergence and somewhat more error, but, overall, the presence of such agents reduced PES. Groups with more high-C_i_ agents were faster but more error-prone, with no net impact on PES. These results are consistent with BDAT predictions.

**Table 5:** Correlations between agent type percentage and performance outcomes.

Agent Type	Ticks	Error	PES
Low-Ci	1.00	−0.72	0.52
Mid-Ci	−0.47	0.23	−0.38
High-Ci	−0.49	0.4	−0.12

Compositions that included more low C_i_ particles were slow, with less error. Compositions that included more high C_i_ particles were fast, but error prone.

We next revisited Table 2 means as a function of BDAT rankings. For ticks, BDAT predicts a rank ordering as follows: High (fastest) > Mid > Mixed-U > Mixed > Polar > Low. The correlation between BDAT rankings and tick-based means was *r* = 0.93, *p* < 0.001, confirming expectations. BDAT predicts that Mixed-U would achieve the lowest error, followed by Mixed > Polar = Low > Mid > High. The correlation between BDAT rankings and error-based means was *r* = 0.93, *p* = 0.009. BDAT rankings for PES were Mixed-U > Mixed > Polar > Mid > High = Low. This correlation of rankings with performance was *r* = 0.91, *p* = 0.013. At least in a simulated environment and when using highly aggregated data, these correlations are impressive.

Recall that in the polarized condition, we counted the number of low- and high-C_i_ agents that were within the convergence zone when using a 50% threshold (100 simulations total). These figures averaged slightly more than 22 (high-C_i_ agents) and slightly less than 3 (low-C_i_ agents), but we will use the numbers 22 and 3 for computational purposes. A 22/3 split departs from 12.5/12.5 expectation, χ^2^ = 14.44, *p* < 0.001, indicating that high-C_i_ agents are more likely to converge around a gBest solution more quickly, consistent with BDAT assumptions.

## Discussion

The current study tested Bounded Diversity Advantage Theory (BDAT, formerly called diversity advantage theory: Pringle and Robinson, 2024) using a novel variant of Particle Swam Optimization (PSO) to model bounded diversity in conscientiousness-like behavior, operationalized as industriousness and persistence through adaptive inertia among swarm agents, with swarm intelligence outcomes operationalized in terms of collective performance (PES) under explicit agreement thresholds. We view PSO as an ABM testbed for decentralized collective problem solving, not as a literal team communication model, and we define outcomes using agreement-sensitive process indicators and speed–accuracy performance metrics that map onto group-process constructs. With respect to this operationalization, the results were broadly consistent with BDAT’s contention that bounded personality diversity should tend to benefit collective intelligence. Diverse compositions (Mixed, Mixed-U, Polar) generally outperformed non-diverse ones (High, Mid, Low) in Performance Efficiency (PES), which balances considerations related to group-level speed and accuracy. Non-diverse compositions were prone to extremes (e.g., groups dominated by high-C_i_ agents converged quickly, but with a pronounced error cost). Moreover, correlational (Table 5) and sorting analyses (Figure 4) revealed that low-C_i_ agents minimized error but increased iterations, while high-C_i_ agents minimized iterations but increased error, with mid-C_i_ agents balancing the two. Diverse mixtures of all three agent types led to the best performance, consistent with a central prediction of BDAT.

From a process-based perspective, the results highlighted an important asymmetry. In the polarized group, approximately 88% of agents in the convergence zone at the 50% threshold were high-C_i_, indicating that low-C_i_ agents delay convergence, which fosters explorations and drives the swarm to more accurate solutions. Other results, such as correlations between BDAT-assigned ranks and overall performance, also displayed patterns consistent with BDAT-based predictions. In the PSO space at least, it seems safe to say that industriousness pushes for group closure and the lack of it fosters group “deliberation” that can be useful with respect to better solutions (Kruglanski et al., 2006). Too many low-C_i_ agents, however, results in convergence times that might be deemed unnecessarily slow.

These findings bridge personality psychology and group process areas, suggesting that bounded diversity along trait dimensions may tend to mitigate premature closure on suboptimal solutions, as seen in the superior PES of diverse compositions. The results align with evolutionary perspectives, where dual-inheritance dynamics (sociobiological mechanisms and sociocultural norms) foster diversity for adaptive group outcomes (Richerson et al., 2016). Such results, and their rationale, extend the Niche Diversity hypothesis (Smaldino et al., 2019) and Coral Reef model (Figueredo et al., 2005) to group-level benefits that occur when there is a diversity of individual niches. From a hierarchical perspective, personality traits can be viewed as integrating, in an emergent manner, bottom-up mechanistic forces (genes in environments, psychological mechanisms) with top-down cultural forces (niches/narratives, norms/functions), where developmental and dual-inheritance evolutionary processes interact via feed-forward, entrainment, and feedback loops. This analysis reframes traits as partly group-based in function, potentially with some facets orthogonal to individual processes, and it encourages refining trait-based taxonomies by distinguishing facets functional to group-level processes.

### Search functions as problem/solution topologies

In complex search functions such as Rastrigin, diverse compositions particularly excelled, suggesting that personality diversity may be particularly valuable in complex decision spaces, analogous to Surowiecki’s (2005) trade-off dilemmas. Simpler functions (e.g., Sphere) sometimes favored non-diverse compositions, indicating that diversity advantages increase with environmental complexity. Across all search functions, diversity benefits persisted across convergence thresholds, though non-diverse high- and mid-C_i_ groups improved slightly at higher thresholds (e.g., 90–100%), as error levels approximated true optima for many search functions.

Examining Figure 5 reveals interesting insights. Top performing (Mixed-U and Mixed), and poorly performing (e.g., Low) compositions were consistent across functions, but the Mid, High, and Polar compositions exhibited more variability. Arguably the simplest function is Sphere, which when manifest as a 2D function with the third dimension being error, resembles a parabolic bowl shape, with smooth, consistent gradients and no local minima. Sphere is an outlier in that it is the only function where the group composition High displayed the best performance. As a problem/solution topology, Sphere is akin to a group decision-making situation where the situation is clear and the prospects of various decisions being considered are unambiguous (e.g., the problem is well understood, and the potential solutions are easy to judge). This is precisely the well-defined, low uncertainty niche situation where BDAT argues that groups of all high-C_i_ agents should tend to perform well. The world is often complex, however, and using a wide variety of search functions better approximates the real world of group decision-making under many circumstances, complex as well as simple.

Studies using PSO with only one search function should be viewed with Figure 5 in mind. Some studies that combine PSO with evolutionary algorithms can be quite computationally demanding, making it more challenging to incorporate multiple search functions. Even so, Figure 5 makes it clear that statements of generalizability would be better served when a variety of search functions are employed.

### Methodological contributions

We should highlight several other methodological innovations. Traditional PSO metrics emphasize global best (gBest) without modeling agreement dynamics, limiting relevance to collective cognition. Our novel convergence zone monitoring tracked spatial clustering around gBest within a definable radius zone, simulating group deliberation reaching agreement at flexible thresholds. The creation of the PES metric was another innovation that handles speed-accuracy tradeoffs, enabling cross-function comparisons via z-scoring to standardize disparate error scales. Adaptive inertia was used to operationalize conscientiousness by modulating momentum based on short-term fitness changes, with high-C_i_ agents maintaining steady effort (*ω* = 0.6) and low-C_i_ agents responding to more momentary successes and failures (±0.3). Agent tracking in Polar compositions supported the expectation that high-C_i_ agents would converge quickly and low-C_i_ agents would drive deliberation, in part due to their “meandering.”

Overall, the present contributions extend PSO’s utility for computational social science, allowing simulation of diverse tasks (e.g., disjunctive, conjunctive, compensatory, Steiner, 1972) and phenomena like consensus-building or information cascades. Reinterpreting search functions as decision-making landscapes further enabled conclusions to be made with respect to a variety of group decision-making landscapes. The choice of 50 agents balanced computational tractability with sufficient swarm size to model stable group-level convergence dynamics while reducing simulation noise.

### Scholarly positioning and scope of extension

The current work bridges three research traditions: (1) computational social science and ABM, where explicit mechanism specification, controlled environments, and bounded claims are central; (2) contemporary swarm intelligence research, where PSO functions as a mature optimization family; and (3) group-process and collective-intelligence research, where process–outcome distinctions and constructs such as agreement, deliberation, and closure are central.

Recent work has increasingly used computational models and agent-based simulations to formalize and test theories of team and group dynamics (e.g., Grand et al., 2016) and recent reviews have mapped how small groups are being modeled in the computational sciences, highlighting opportunities for tighter construct alignment between theory and computational implementations (e.g., Jackson, 2025; Lehmann-Willenbrock et al., 2017). Adjacent traditions further motivated our approach. In evolutionary computation, diversity-preserving “niching” methods (e.g., fitness sharing and crowding) were developed to mitigate premature convergence in multimodal search spaces. Such work (e.g., Goldberg and Richardson, 1987; Mahfoud and Mani, 1996) can be viewed as a conceptual parallel to BDAT’s emphasis on bounded diversity as a mechanism for avoiding premature closure, though our implementation focus was particular to PSO and did not involve comparison across optimization families. In parallel, contemporary empirical work on collective intelligence provides measurement targets and motivating evidence for group-level performance differences (e.g., Riedl et al., 2021). Our contribution is complementary in using a controlled agent-based testbed to probe a bounded subset of mechanisms relevant to those outcomes rather than attempting to model human conversational teams directly.

In this interdisciplinary framing, our contribution could be considered circumscribed. We did not treat PSO as a literal model of conversational teams, but as an ABM testbed for a subset of BDAT mechanisms that can be instantiated in terms of decentralized information sharing, convergence dynamics, and bounded behavioral variation. With respect to this scholarly alignment perspective, we extended BDAT by providing a computational operationalization of its bounded-diversity prediction within a swarm-like testbed. We also extended PSO as a modeling formalism (rather than as a competitive benchmark contribution) by adding agreement-sensitive monitoring and a speed–accuracy outcome metric. And we extended group-process interpretations by distinguishing the best-found solution from the degree to which the collective adopts it under explicit agreement thresholds.

### PSO and other FFM traits

PSO was developed to empirically outperform alternative swarm intelligence optimization algorithms. This history of development could be considered to be consistent with the present operationalization of BDAT-derived predictions with respect to the FFM trait dimension of agreeableness because bounded diversity in agreeableness-like behavior is already present in the canonical PSO. Specifically, the coefficients r_1_c_1_ and r_2_c_2_ impart a diversity of prosocial-like behavior in the swarm through the injection of randomness from r_1_ and r_2_, which effectively changes the weighting impact of c_1_ and c_2_. According to this mapping, highly agreeable agents favor gBest (r_2_c_2_ > r_1_c_1_), giving deference to what the group wants, while disagreeable agents are created when r_1_c_1_ > r_2_c_2_, which results in prioritizing personal preference (pBest) over group preference. PSO variants that lack this agreeableness-like diversity (no randomness inserted) or that are tuned with asymmetric mean coefficients (c_1_ ≠ c_2_) across the swarm perform worse than canonical PSO over the breadth of functions and typically are only superior for some application-specific functions. In preliminary explorations, we systematically manipulated these asymmetries and found that compositions with diversity in agreeableness-related preferences outperform compositions that lack agent-based diversity in such preferences.

The few other attempts to operationalize FFM traits with PSO (e.g., Lim et al., 2023) have typically been limited to one search function (e.g., parabola), which, according to Figure 5, could limit the generalizability of the relevant results. Additionally, these prior approaches have typically used gBest as the sole performance measure, limiting the type of task being simulated to disjunctive tasks, where the performance of the group is based on the performance of its best member. From our perspective, tracking gBest as the only outcome requires the assumption that the group will adopt gBest in a manner that is not included in the modeling itself. Our approach, instead, is capable of measuring agreement dynamics, as defined by convergence of a certain percentage of agents, which enables the use of PSO as an agent-based modeling approach that is inclusive of disjunctive (the best individual outcome), conjunctive (all individuals must put forth effort), compensatory (average across individuals), additive (sum of individual efforts), and discretionary (group determines how to combine efforts) tasks (Steiner, 1972) as well as various decision-making rules (e.g., reaching majority, finding consensus, or achieving unanimity).

### Limitations and practical implications

Limitations to our study include the narrow operationalization of conscientiousness, capturing only elements of industriousness/persistence and omitting facets like orderliness. Parameter choices (e.g., *ω* = 0.6 baseline) could be quibbled with. Some could challenge our assertion that search topologies are akin to decision-making situations or that embodied cognition maps best to 2D spaces with a third dimension for error. Convergence zones assume spatial proximity equates to agreement, which can be criticized for oversimplifying human communication. The lack of real-world validation positions the work in terms of theoretical foundations rather than prescriptive guides, with the need for laboratory and/or field studies that attend to personality diversity (rather than mean personality) as a basis for group performance.

While we have started the process of using PSO to test BDAT, future research should operationalize other facets and traits (e.g., neuroticism facets via avoidance parameters) and test dynamic topologies (search functions that change shape with ticks) to simulate evolving decision-making landscapes or competitive scenarios. Although more research is needed, our findings challenge viewing conscientiousness as uniformly positive as lower levels of conscientiousness (“laziness”) among some group members may prevent groupthink and enhance innovation in complex tasks. For organizations, diverse conscientiousness profiles may optimize teams in some decision-making contexts, which could lead to reevaluating hiring to include more variety in how tasks are approached by individuals. In policy, including fewer consensus-driven members could yield better outcomes, though at the cost of the added deliberation time.

As a final note, the current study did not attempt to evaluate the full breadth of contemporary group diversity and discourse traditions (e.g., diversity in interaction networks, team role structure, or modern conversational groupthink paradigms). Rather, consistent with our scholarly positioning, PSO was used as an ABM testbed to instantiate a swarm-like subset of BDAT mechanisms – bounded behavioral variation, decentralized information sharing, and convergence/closure dynamics – and our inferences are limited to such instantiations.

## Conclusion

The current results are illuminating in supporting the idea that bounded diversity in something like personality (i.e., individual differences in agent preferences and behaviors) can boost swarm intelligence, here measured as collective problem-solving performance (speed and accuracy with respect to agreement thresholds). Although the present results do not by themselves validate BDAT (Pringle and Robinson, 2024) in its full theoretical scope, they do suggest that the theory is tractable enough to generate computationally testable predictions, therefore meriting further examination across alternative operationalizations and human-group contexts. The results also speak to the integration of personality, evolutionary, and group-related perspectives, laying the groundwork for further investigations of personality diversity’s role in collective intelligence.

## Data Availability

The raw data supporting the conclusions of this article will be made available by the authors, without undue reservation.
